# Role of dexamethasone dosage in combination with 5-HT_3_ antagonists for prophylaxis of acute chemotherapy-induced nausea and vomiting

**DOI:** 10.1038/sj.bjc.6690100

**Published:** 1999-02

**Authors:** K Münstedt, H Müller, E Blauth-Eckmeyer, K Stenger, M Zygmunt, H Vahrson

**Affiliations:** Department of Gynecological Oncology and Radiotherapy, Justus-Liebig-University Giessen, Klinikstrasse 32, Giessen, D 35392, Germany; Synthelabo Arzneimittel, Lindberghstrasse 1, Puchheim, 82178, Germany

**Keywords:** nausea, emesis, vomiting, corticosteroid, dexamethasone

## Abstract

Dexamethasone (20 mg) or its equivalent in combination with 5-HT_3_ antagonists appears to be the gold-standard dose for antiemetic prophylaxis. Additional to concerns about the use of corticosteroids with respect to enhanced tumour growth or impaired killing of the tumour cells, there is evidence that high-dosage dexamethasone impairs the control of delayed nausea and emesis, whereas lower doses appear more beneficial. To come closer to the most adequate dose, we started a prospective, single-blind, randomized trial investigating additional dosage of 8 or 20 mg dexamethasone to tropisetron (Navoban), a 5-HT_3_ receptor antagonist, in *cis*-platinum-containing chemotherapy. After an interim analysis of 121 courses of chemotherapy in 69 patients, we have been unable to detect major differences between both treatment alternatives. High-dose dexamethasone (20 mg) had no advantage over medium-dose dexamethasone with respect to objective and subjective parameters of acute and delayed nausea and vomiting. In relation to concerns about the use of corticosteroids in non-haematological cancer chemotherapy, we suggest that 8 mg or its equivalent should be used in combination with 5-HT_3_ antagonists until further research proves otherwise. © 1999 Cancer Research Campaign


*British Journal of Cancer* (1999) **79**(3/4), 637–639
© 1999 Cancer Research Campaign
Article no. bjoc.1998.0100

### Role of dexamethasone dosage in combination with 5-HT$_3$ antagonists for prophylaxis of acute chemotherapy-induced nausea and vomiting

**K Münstedt[1], H Müller[1], E Blauth-Eckmeyer[2], K Stenger[1], M Zygmunt[1] and H Vahrson[1]**

[1]Department of Gynecological Oncology and Radiotherapy, Justus-Liebig-University Giessen, Klinikstrasse 32, D 35392 Giessen, Germany; [2]Synthelabo Arzneimittel, Lindberghstrasse 1, 82178 Puchheim, Germany

**Summary** Dexamethasone (20 mg) or its equivalent in combination with 5-HT$_3$ antagonists appears to be the gold-standard dose for antiemetic prophylaxis. Additional to concerns about the use of corticosteroids with respect to enhanced tumour growth or impaired killing of the tumour cells, there is evidence that high-dosage dexamethasone impairs the control of delayed nausea and emesis, whereas lower doses appear more beneficial. To come closer to the most adequate dose, we started a prospective, single-blind, randomized trial investigating additional dosage of 8 or 20 mg dexamethasone to tropisetron (Navoban), a 5-HT$_3$ receptor antagonist, in *cis*-platinum-containing chemotherapy. After an interim analysis of 121 courses of chemotherapy in 69 patients, we have been unable to detect major differences between both treatment alternatives. High-dose dexamethasone (20 mg) had no advantage over medium-dose dexamethasone with respect to objective and subjective parameters of acute and delayed nausea and vomiting. In relation to concerns about the use of corticosteroids in non-haematological cancer chemotherapy, we suggest that 8 mg or its equivalent should be used in combination with 5-HT$_3$ antagonists until further research proves otherwise.

**Keywords:** nausea; emesis; vomiting; corticosteroid; dexamethasone

Corticosteroids exert antiemetic effects as single drugs and enhance the effects of other antiemetics such as benzamides and 5-HT$_3$ receptor antagonists (Blazon and Zorzano-Estrade, 1987; Hawthorn and Cunningham, 1990; Stephens et al, 1990). The mechanism of action whereby corticosteroids affect nausea is not understood. Possible mechanisms of action have been suggested:

- modified capillary permeability of the CNS and reduced exposure of emetic trigger sites to toxic stimuli (Livera et al, 1985);
- reduced levels of 5-HT in neural tissue by depletion of its precursor, tryptophan (Young, 1981);
- the anti-inflammatory properties of cortisol may prevent the release of serotonin in the gut or prevent activation of 5-HT receptors in the gastrointestinal system (Fredrikson et al, 1992);
- sensitization of the 5-HT$_3$ receptor (Sagar, 1991).

For antiemetic prophylaxis, 20 mg of dexamethasone or its equivalent appears to be the gold-standard dose and most commonly used in routine antiemetic prophylaxis as well as clinical trials. However, concern about the use of corticosteroids has been expressed because of the possibility of enhanced tumour growth (Haid, 1981), higher incidence of distant tumour (Sherlock and Hartmann, 1962), or impaired killing of the tumour cells (Powell et al, 1990). In in vitro experiments, the addition of corticosteroids did not reduce the anti-tumoral effect of cisplatinum (Aapro and Alberts, 1981). In contrast, corticosteroids are important components of many antineoplastic regimens, especially haematological malignancies, or are required to reduce the side-effects of some cytotoxic agents such as taxanes. But the problem still remains unsolved.

To the best of our knowledge, there have been no earlier investigations on the importance of dexamethasone dosage in antiemetic therapy. Earlier studies have mainly investigated different corticosteroid quantities in comparison with no or placebo medication. Prior work in this area suggests that especially patients with low prechemotherapy night-time cortisol excretion profit from cortisol administration (Fredrickson et al, 1992), that endogenous cortisol exerts antiemetic effects similar to that of exogenous corticosteroids (Hursti et al, 1993), and dexamethasone in high doses (20 mg) may impair the control of delayed symptoms (Peterson et al, 1996) whereas this adverse effect was not observed at doses of 8 mg (Carmichael et al, 1994).

To study benefits and side-effects of single high-dose dexamethasone (20 mg) in comparison to single medium-dose dexamethasone (8 mg), we started this prospective, randomized, single-blind trial. Satisfactory antiemetic control was also observed with 8 mg dexamethasone in other trials.

### PATIENTS AND METHODS

Sixty-nine patients assigned to receive either PEC (*cis*-platinum 50 mg m$^{-2}$, epirubicin 60 mg m$^{-2}$ and cyclophosphamide 500 mg m$^{-2}$) or PTx (*cis*-platinum 75 mg m$^{-2}$ and paclitaxel 175 mg m$^{-2}$) combination chemotherapy on 1 day in the first line on an in-patient basis upon histologically confirmed ovarian cancer were enrolled in this single-blind, prospective trial. Patients with CNS

*Received 4 February 1998*
*Revised 29 May 1998*
*Accepted 9 June 1998*

*Correspondence to:* K Münstedt

**Table 1** Criteria for inclusion and exclusion

| |
|---|
| Criteria for inclusion in this study |
| Histologically proven ovarian cancer, abdominal carcinoma, or cancer of the fallopian tube |
| Treatment with PEC or PTx – combination chemotherapy |
| Informed consent |
| Criteria for exclusion |
| CNS metastases |
| Malfunction of liver, kidney or heart |
| Subileus or ileus in patients' history |
| Allergy against 5-$HT_3$ antagonists and/or alizapride |
| Drug and alcohol abuse |
| Nausea and emesis with no correlation to chemotherapy |
| Anticipatory nausea and vomiting |
| More than five vomiting episodes in a preceding cycle of chemotherapy |
| Concomitant corticosteroid medication |

metastases, malfunction of liver, kidney or heart, subileus or ileus in their history, allergy against 5-$HT_3$ antagonists and/or alizapride, drug and alcohol abuse, nausea and emesis with no correlation to chemotherapy, anticipatory nausea and vomiting, more than five vomiting episodes in a preceding cycle of chemotherapy, and concomitant corticosteroid medication were primarily excluded from the trial. Patients' characteristics are listed in Table 1. In case of patients who were not chemotherapy naive, all of them had 20 mg of dexamethasone on the same chemotherapy protocol as before. Patients ($n = 52$) were allowed to participate in the study twice, receiving the opposite alternative of antiemetic prophylaxis at the second time. Seventeen patients received only one course of chemotherapy, either because of failure of the antiemetic prophylaxis or end or change of chemotherapy. Stratification was carried out using a randomization list according to planned chemotherapy, the number of preceding chemotherapy courses and experiences with nausea and emesis (e.g. hyperemesis gravidarum). The alternatives for prophylaxis of acute nausea and vomiting were: (a) dexamethasone (Fortecortin, Merck, Germany; 20 mg) plus tropisetron (Navoban, Novartis, Germany; 5 mg); or (b) dexamethasone (8 mg) plus tropisetron (5 mg).

Patients were allowed normal food and drink intake before chemotherapy. Beginning at about 08:00 h, patients received 1 l of PÄD-II solution (Fresenius, Germany) intravenously until either (a) or (b) antiemetic prophylaxis was given 30 min before chemotherapy at about 12:00 h. Food intake was not permitted until 8 h after chemotherapy. Until the next morning, all patients received an additional 1 l of PÄD-II solution and 2 l of saline intravenously. In case of five or more vomiting episodes on the day of chemotherapy, patients received triflupromazine suppositories (70 mg, Psyquil, Sanifi Winthrop) as a rescue medication. These patients were excluded from participation in the study a second time.

During days 2–4, all patients received alizapride (Vergentan, Synthelabo, Germany; $3 \times 100$ mg) orally for prophylaxis of delayed nausea and vomiting, which has proven efficacy (Münstedt et al, 1995). During their stay in the hospital over the entire study period, patients completed a self-report diary daily, in which the number of vomiting and retching episodes were recorded. A retching or vomiting episode was defined to have ended when at least 1 min had passed since retching or vomiting ceased. Wellbeing, including nausea, was assessed on the same basis using a translated version of the Rotterdam Symptom Checklist (de Haes et al, 1990).

**Table 2** Patients' characteristics

| | Group A (20 mg) (%) | Group B (8 mg) (%) |
|---|---|---|
| Age | | |
| <40 | 1.9 | 0 |
| 41–50 | 9.3 | 11.9 |
| 51–60 | 18.5 | 22.4 |
| 61–70 | 59.3 | 47.8 |
| >70 | 11.1 | 17.9 |
| FIGO stage | | |
| I | 14.8 | 19.4 |
| II | 18.5 | 19.4 |
| III | 51.9 | 46.3 |
| IV | 14.8 | 14.9 |
| Preceding courses of chemotherapy | | |
| 0 | 40.2 | 40.3 |
| 1 | 27.3 | 28.4 |
| <4 | 20.0 | 22.4 |
| <7 | 12.5 | 8.9 |

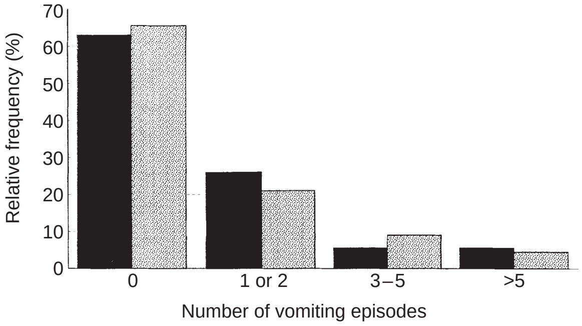

**Figure 1** Relative frequency of number of vomiting episodes. Complete response (0 vomiting episodes); major control (1 or 2 vomiting episodes); minor control (3–5 vomiting episodes); or failure (>5 vomiting episodes). Group A (20 mg dexamethasone, ■); group B (8 mg dexamethasone, ▨)

Data analyses were carried out using the Wilcoxon and Mann–Whitney *U*-tests; and Kruskal–Wallis analysis with the help of the testimate 5.1 and SPSS for Windows 7.0 computer programs.

### RESULTS

### Objective parameters

No significant differences were observed between both groups during the observation period from day 0 to day 5 with respect to objective parameters, which included absolute number of vomiting and retching episodes and their distribution during the day, bowel movement and food intake. Figure 1 shows the similar distribution of objective vomiting on the day of chemotherapy in both groups.

### Subjective parameters

Either treatment alternative of 20 mg or of 8 mg dexamethasone had no statistically significant influence on subjective parameters, including nausea, of the Rotterdam Symptom Checklist (de Haes et al, 1990) during the observation period. Both antiemetic regimens were well tolerated. Of the patients in group A, 64.8% compared with 82.1% of group B considered efficacy and tolerability of the antiemetic prophylaxis to be good or very good. Of group A patients,

 *© Cancer Research Campaign 1999*

75.9% expressed a desire to receive the same medication in a potential next course of chemotherapy compared with 82.1% in group B.

Because this interim analysis after 121 courses of chemotherapy failed to show a major benefit of either dexamethasone dose, we decided to discontinue the study because of the lack of later clinical relevance of the results. We believe that, for a final conclusion on the subject, a prospective, randomized, double-blinded study would be more appropriate.

### DISCUSSION

To the best of our knowledge, there have been no earlier trials evaluating the importance of dexamethasone dosage in antiemetic prophylaxis. Trials using either dose have been published (Campora et al, 1994; Carmichael et al, 1994). The benefit of additional corticosteroid therapy to 5-$HT_3$ antagonists is undisputed. As already mentioned above, there is controversy about the safety of corticosterid administration as an antiemetic substance in cancer chemotherapy in non-haematological malignancies in which corticosteroids are not generally administered.

This study failed to prove a clinical advantage of high-dose dexamethasone (20 mg) over medium-dose dexamethasone (8 mg) in combination with tropisetron for prophylaxis of acute nausea and vomiting. Referring to the work of Peterson et al (1996), we were also unable to detect differences between the control of delayed symptoms. Perhaps impaired control of delayed symptoms is a sequel of corticosteroid administration for prophylaxis of acute nausea and vomiting.

As a compromise between unknown risks and proven benefits, we believe that corticosteroid usage should be handled reluctantly, which applies to both the use in general as well as the required doses. The lowest effective corticosteroid dose should be chosen until further studies provide more knowledge on this topic. Doses equivalent to 8 mg dexamethasone seem to be appropriate at the present time because this study failed to demonstrate a significant clinically relevant difference between high and moderate dexamethasone treatment. Perhaps even smaller doses may be sufficient, which will be a topic of future investigations.

### ACKNOWLEDGEMENTS

This study was supported by Novartis Pharmaceutical Company. We are indebted to Dr Snipes of the Institute of Anatomy of the Justus-Liebig-University, Giessen, for linguistic revision of the manuscript.

### REFERENCES

Aapro MS and Alberts DS (1981) High-dose dexamethasone for prevention of cisplatin-induced vomiting. *Cancer Chemother Pharmacol* **7**: 11–14

Balzon J-C and Zorzano-Estrade C (1987) Utilisation de l'alizapride (Plitican) seul ou associé à une forte dose de corticoides dans les troubles digestifs induits par le cisplatine. *Comptes Rendus Ther Pharmacol Clin* **52**: 13–19

Campora E, Giudici S, Merlini L Rubagotti A and Rosso R (1994) Ondansetron and dexamethasone versus standard combination antiemetic therapy. *Am J Clin Oncol* **17**: 522–526

Carmichael J, Bessel EM, Harris AL, Hucheon AW, Dawes PJDK and Daniels S (1994) Comparison of granisetrone alone and granisetron plus dexamethasone in the prophylaxis of cytotoxic-induced emesis. *Br J Cancer* **70**: 1161–1164

de Haes JCJM, van Knippenberg FCE and Neijt JP (1990) Measuring psychological and physical distress in cancer patients: structure and application of the Rotterdam symptom checklist. *Br J Cancer* **62**: 1034–1038

Fredrikson M, Hursti T, Fürst CJ, Steineck G, Börjeson S, Wikblom M and Peterson C (1992) Nausea in cancer chemotherapy is inversely related to urinary cortisol excretion. *Br J Cancer* **65**: 779–780

Haid M (1981) Steroid antiemesis may be harmful. *N Engl J Med* **304**: 1237

Hawthorn J and Cunningham D (1990) Dexamethasone can potentiate the antiemetic action of a 5-$HT_3$-receptor antagonist on cyclophosphamide induced vomiting in the ferret. *Br J Cancer* **61**: 56–60

Hursti TJ, Fredrikson M, Steineck G, Börjeson S, Fürst CJ and Peterson C (1993) Endogenous cortisol exerts antiemetic effect similar to that of exogenous corticosteroids. *Br J Cancer* **68**: 112–114

Livera P, Trojano M and Simone I (1985) Acute changes in blood CSF barrier permselectivity to serum proteins after intrathecal methotrexat and CNS irradiation. *J Neurol* **231**: 336–339

Münstedt K, Milch W, Blauth-Eckmeyer E, Spänle A and Vahrson H (1995) Prevention of cisplatinum-induced delayed emesis and nausea. *Onkologie* **18**: 23–26

Peterson C, Hursti TJ, Börjeson S, Avall-Lundquist E, Fredrickson M, Fürst CJ, Lomberg H and Steineck G (1996) Single high-dose dexamethasone improves the effect of ondansetron on acute chemotherapy-induced nausea and vomiting but impairs the control of delayed symptoms. *Support Care Cancer* **4**: 440–446

Powell CB, Mutch DG, Kao M-S, Wen-Yun J, Perry DL, Westphale E and Collins JL (1990) Dexamethasone used as an antiemetic in chemotherapy protocols inhibits natural cytotoxic (NC) cell activity. *Cancer* **65**: 466–472

Sagar S (1991) The current role of anti-emetic drugs in oncology: a recent revolution in patients' symptom control. *Cancer Treat Rev* **18**: 95–135

Sherlock M and Hartmann WH (1962) Adrenal steroids and the pattern of metastases of breast cancer. *JAMA* **181**: 313–317

Stephens SH, Silvey VL and Wheeler RH (1990) A randomized, double-blind comparison of the antiemetic effect of metoclopramide and lorazepam with or without dexamethasone in patients receiving high-dose cisplatin. *Cancer* **66**: 443–446

Young SN (1981) Mechanism of decline in rat brain 5-hydroxytryptamine after induction of liver tryptophan pyrrolase by hydrocortisone: roles of tryptophan catabolism and kynurenine synthesis. *Br J Pharmacol* **74**: 695

© Cancer Research Campaign 1999
